# Antioxidant and Anti-Apoptotic Neuroprotective Effects of Cinnamon in Imiquimod-Induced Lupus

**DOI:** 10.3390/antiox13070880

**Published:** 2024-07-22

**Authors:** Georges Maalouly, Christine-Marie-Anne Martin, Yara Baz, Youakim Saliba, Anna-Maria Baramili, Nassim Fares

**Affiliations:** Laboratory of Research in Physiology and Pathophysiology, Pole of Technology and Health, Faculty of Medicine, Saint Joseph University of Beirut, Beirut 1104 2020, Lebanon; georges.maalouly@usj.edu.lb (G.M.); christine-marie-anne.martin@net.usj.edu.lb (C.-M.-A.M.); yara.baz@net.usj.edu.lb (Y.B.); youakim.saliba@usj.edu.lb (Y.S.); anna-maria.baramili1@usj.edu.lb (A.-M.B.)

**Keywords:** lupus, brain, cinnamon, oxidative stress, apoptosis

## Abstract

Background: Despite accumulating evidence correlating oxidative stress with lupus disease activity, the brain redox pathways are still poorly investigated. Cinnamomum cassia, a widely used spice with powerful antioxidant properties, could be a novel therapeutic candidate in lupus. Methods: C57BL/6J female mice were divided into five groups: sham, sham-cinnamon, lupus, lupus-cinnamon starting from induction, and lupus-cinnamon starting two weeks before induction. Lupus was induced by skin application on the right ear with 1.25 mg of 5% imiquimod cream three times per week for six weeks. Cinnamomum cassia was given orally, five days per week, at 200 mg/kg. Results: Concomitant to TLR7-MYD88 pathway activation, the p-NRF2/NRF2 and p-FOXO3/FOXO3 ratios were increased in the hippocampus and alleviated by cinnamon treatment. BCL-2 positivity was enhanced in hippocampal neurons and reversed only by preventive cinnamon administration. In vitro, exposure of hippocampal cells to the plasma of different groups induced a surge in oxidative stress. This was associated with an increased t-BID/BID ratio. Cinnamon treatment, particularly in the preventive arm, normalized these modifications. Conclusions: Our study shows a neuroprotective effect of cinnamon by rescuing brain redox and apoptosis homeostasis in lupus, paving the way for its use as a natural therapeutic compound in the clinical management of lupus.

## 1. Introduction

Systemic lupus erythematosus (SLE) is a prototype of autoimmune diseases. Its pathophysiology is complex and its management is mainly immunosuppressive. Genetic and epigenetic factors contribute to the formation of immune complexes and organ damage. Recently, accumulating evidence suggests that oxidative stress is involved in disease activity and tissue damage [1].

Excessive generation of reactive oxygen species (ROS), with incomplete clearance, seems to participate in the initiation of SLE pathogenesis [2]. Oxidative stress contributes to endogenous antigen exposure and enhances apoptotic signals in SLE [3].

ROS levels are increased in all tissues of *MRL/lpr* mice, suggesting that the pathologic abnormalities in SLE mice correlate with enhanced oxidative stress [4]. The brain, an organ with elevated oxygen consumption, is highly vulnerable to peroxidation. Thus, the neurological pathologies related to disrupted redox homeostasis are associated with the upregulation of reactive mechanisms such as nuclear factor E2-related factor 2 (Nrf2) pathway activation and astrogliosis [5]. However, the modification of cerebral redox-sensitive pathways in lupus animal models awaits further investigation. In a recent study of spontaneous lupus in *MRL/lpr* mice, cerebral tissue was the most critically affected by oxidative damage, with lower mRNA expression of mitochondrially encoded cytochrome c oxidase 1 (MT-CO1) and higher malondialdehyde (MDA) levels [4]. Dysregulated redox homeostasis was also retrieved in the brains of MRL/*lpr* mice, with activation of protective pathways (astrocyte and and astrocyte activation) [5]. This disruption was age-dependent and reversed by conjugated linoleic acid supplementation [5]. Additionally, accumulating data suggest that oxidative stress may induce apoptosis via both mitochondria-dependent and mitochondria-independent pathways in different normal and pathological situations [6]. Recently, neuropsychiatric lupus was documented in an experimental model using imiquimod, a TLR7 agonist [7]. Although new data highlight the role of the TLR7–MyD88 axis in lupus [8] and its interaction with oxidative stress in the brain tissues [9] of spontaneous lupus models, targeting this axis with antioxidants in induced neuropsychiatric lupus models has not been investigated.

Antioxidant agents constitute a novel preventive and therapeutic strategy in balancing oxidation–antioxidation status. Recently, neuroprotective natural compounds with ROS-scavenging properties are receiving more attention. Their neuroprotective effects modulate tissue damage and apoptosis by acting on different oxidative pathways [10].

*Cinnamomum cassia* bark, a widely used flavoring spice, exhibits multiple pharmacological effects [11]. Numerous studies have indicated that cinnamon contributes to antioxidant protection by decreasing MDA levels and enhancing antioxidant enzyme activities [12,13]. Cinnamaldehyde, one of the active compounds of cinnamon, has the electrophilic ability to modulate cellular redox homeostasis [14]. The significant therapeutic role of cinnamon polyphenols in traumatic brain injury is related to the suppression of oxidative injury and inflammation [15]. In an acetaminophen overdose model, cinnamon oil ameliorated the antioxidant status and regulated cytokines in cerebral tissue [16].

In this study, we aim to evaluate the neuroprotective potential of cinnamon to alleviate brain oxidative stress and apoptosis in imiquimod-induced lupus.

## 2. Materials and Methods

### 2.1. Ethical Statement

This study was approved by the Ethical Committee of Saint Joseph University (CEHDF 1762, 2 December 2020) and adhered to established animal care protocols. It followed the Guiding Principles in the Care and Use of Animals by the American Physiological Society, and the methodologies aligned with the NIH’s “Guide for the Care and Use of Laboratory Animals” (NIH Publication no. 85–23, revised 1996). The study complied with the European Parliament Directive 2010/63 EU, ensuring ethical and humane treatment of animals.

### 2.2. Animals and Study Groups

**Figure 1:**
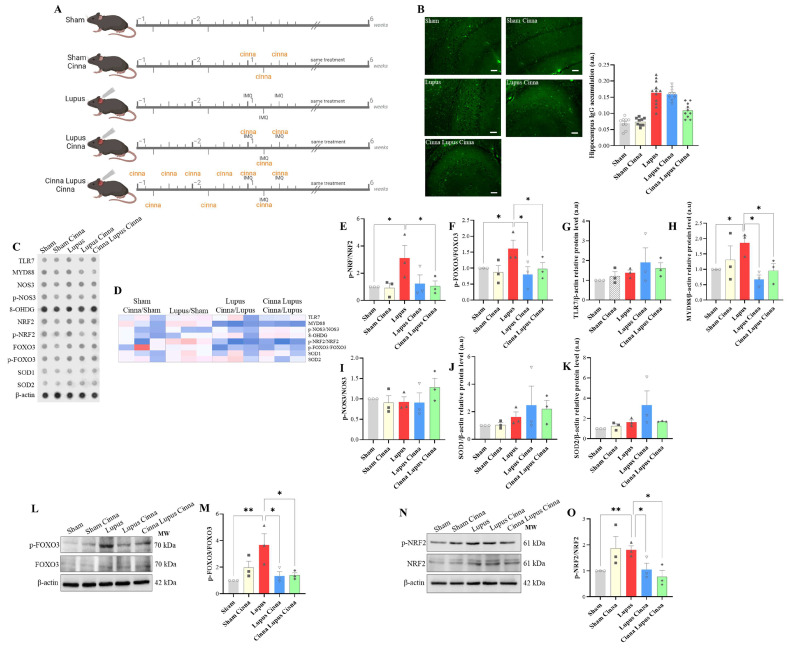
(**A**): Schematic representation of the experimental design, showing imiquimod treatment and cinnamon administration for the different groups: sham, sham treated with cinnamon (Sham Cinna), imiquimod-induced lupus (Lupus), imiquimod-induced lupus with cinnamon treatment (Lupus Cinna), and imiquimod-induced lupus with cinnamon administration starting 2 weeks before induction and continued after induction (Cinna Lupus Cinna). (**B**) Hippocampal immunofluorescence (scale bars 15 μm, magnification ×100) with quantification of IgG accumulation in the different groups. (**C**,**D**) Antibody array (**C**) with heatmap (**D**) of oxidative stress markers, TLR7 and MYD88, for the different groups. (**E**–**K**) Quantification of p-NRF/NRF, p-FOXO3/FOXO3, TLR7/β-actin, MYD88/β-actin, p-NOS3/NOS3, SOD1/β-actin, and SOD2/β-actin. (**L**,**M**) Western blot and quantification of p-FOXO3/FOXO3. (**N**,**O**) Western blot and quantification of p-NRF/NRF. a.u.: arbitrary units. * *p* < 0.05 and ** *p* < 0.01.

Fifty adult female C57BL/6J mice were used in this study. Animals were kept in an environment with a constant temperature of 25 °C and humidity of 50 ± 5%, and they experienced a 12:12 h light–dark cycle. They had access to standard rodent food and tap water ad libitum. Lupus was induced by skin application on the right ear with 1.25 mg of 5% imiquimod cream three times per week for six weeks. *Cinnamomum cassia* powder (Solgar, Leonia, NJ, USA, powder of cinnamon bark and bark extract) was orally administered at 200 mg/kg/day to each animal individually on a small piece of chow slightly moistened with water in order to retain the cinnamon powder. Furthermore, the experimenter verified each time that the mouse had completely ingested this piece of chow. Accordingly, the animals were divided into five groups of ten mice per group: Sham, Sham Cinna, Lupus, Lupus Cinna (LC), and Cinna Lupus Cinna (CLC) (Figure 1A). At the end of the protocol, the animals were anesthetized with a combination of ketamine (75 mg/kg; Interchemie, Waalre, The Netherlands) and xylazine (10 mg/kg; RotexMedica, Trittau, Germany). Once they showed no response to toe pinching, indicating deep anesthesia, they were euthanized for subsequent tissue collection. Blood samples were further collected in EDTA tubes. Plasma was collected after centrifugation at 4500 rpm for 15 min and then stored at −80 °C for later treatment of neural cell cultures. For brain extraction, first the scalp was surgically removed to reveal the front part of the skull. The cranial bone was cut through the cranial sutures, and a scalpel blade was employed to excise the segments of the skull. The connective tissue was then surgically removed, and the brain was extracted from the cranial cavity. The brain was longitudinally cut in half, with one piece conserved in 10% neutral buffered formalin for histological analysis, and the hippocampus was removed from the other piece and kept at −80 °C for later protein extraction.

Regarding the in vitro segment of the study, hippocampal cells were isolated for treatment with imiquimod, cinnamon, and plasma, following the sacrifice of six female C57BL/6J mice.

### 2.3. Hippocampal Cell Culture

Hippocampal cells were isolated as previously described [17,18]. Briefly, the brain was harvested, with removal of the olfactory lobe and cerebellum, followed by a sagittal section at the level of the hippocampus. The hippocampus was carefully extracted and then transferred to modified Tyrode’s solution (in mM): 117 NaCl; 5.7 KCl; 1.7 MgCl_2_; 4.4 NaHCO_3_; 1.5 KH_2_PO_4_; 10 HEPES; 10 creatine monohydrate; 20 taurine; and 11.7 D-glucose; pH 7.1 with NaOH. Tissues were dissected into small ≈2 mm pieces and then digested with two successive 20 min enzymatic baths containing type V collagenase (157 U·mL^−1^). The supernatants from both baths were combined, and the cells were collected after centrifugation at 2000 rpm for 10 min. The cells were cultured in Dulbecco’s Modified Eagle’s Medium (DMEM) enriched with 10% fetal bovine serum, 4 mM L-glutamine, 1 mM sodium pyruvate, and 1% penicillin/streptomycin. All media were from Sigma-Aldrich, St. Louis, MO, USA.

Following five days of culture, the hippocampal cells were treated for 24 h with the plasma (1/1000) extracted from five mice in the previous control and lupus mouse groups. Another series of cells was treated with imiquimod (10 µg/mL) in the presence or absence of cinnamon extract (1/1000).

### 2.4. Hippocampus Histology and Immunofluorescence

Neutral buffered formalin supplemented with sodium dihydrogen phosphate monohydrate (NaH_2_PO_4_·H_2_O) and disodium hydrogen phosphate anhydrous (Na_2_HPO_4_) was used (pH = 7.0). The fixed hippocampal tissue was embedded in paraffin and cut into sections of 4 μm (Sigma-Aldrich). After rinsing the sections with distilled water, the sections were dehydrated in ethanol/water baths with decreasing water content and rinsed with xylene. For antigen retrieval, the sections were incubated with 2N HCl for 20 min at room temperature. This was followed by 20 min incubation with 0.3 M glycine, then 20 min permeabilization with Triton X-100, at room temperature. Finally, blocking was achieved in the presence of 10% goat serum and 1% bovine serum albumin (BSA) diluted in phosphate-buffered saline for 1 h at 37 degrees Celsius. To detect IgG, the sections were incubated with the rabbit anti-mouse IgG H&L Alexa Fluor 594 secondary antibody for 30 min at 37 degrees. Otherwise, anti-BCL2 (ab59348; Abcam, Cambridge, UK) was used followed by goat anti-rabbit H&L Alexa Fluor 488. Finally, the sections were treated with Fluoroshield Mounting Medium containing 4′,6-diamidino-2-phenylindole (DAPI) (Abcam, Cambridge, UK) and pictures were captured using an Axioskop 2 immunofluorescence microscope (Carl Zeiss Microscopy GmbH, Jena, Germany) equipped with a CoolCube 1 CCD camera (MetaSystems, Newton, MA, USA). ImageJ was used for analysis and quantification.

### 2.5. Western Blot and Antibody Array

The isolated hippocampus and the cultured hippocampal cells were homogenized and lysed in assay lysis buffer consisting of NaCl (150 mM), Tris OH pH 7.5 (50 mM), EDTA (95 mM), and Triton X-100 (0.5%) and containing protease and phosphatase inhibitors (cat#22020008, BioWorld, Dublin, OH, USA and Sigma-Aldrich, St. Louis, MO, USA, respectively) for total protein extraction. The Bradford protein assay (Bio-Rad, Marnes-la-Coquette, France) was used for protein concentration measurement. After that, samples were denatured in Laemmli loading buffer (Bio-Rad) containing 10% β-mercaptoethanol (Sigma-Aldrich) at 37 °C for 20 min. Protein separation was achieved via SDS 12% PAGE and transferred onto polyvinylidene fluoride membranes (Bio-Rad) blocked by either 5% non-fat milk or 5% BSA. Overnight membrane incubation was performed with the corresponding primary antibodies at 4 °C: anti-FOXO3 (NBP2-16521), anti-FOXO3 (pSer253) (NBP2-67521), anti-NRF2 (NBP1-32822), anti-NRF2 (pSer40) (NBP2-67465) (Novus Biologicals, Bio-Techne, MN, USA), and anti-BCL2 (ab59348; Abcam, Cambridge, UK). For protein loading control, the blots were re-probed for β-actin (SAB1305554) (Sigma-Aldrich, USA). Goat anti-rabbit and anti-mouse antibodies (1/3000, Bio-Rad Laboratories) were used as secondary antibodies.

Regarding the antibody arrays, dot blot equipment (Cleaver Scientific, Rugby, UK) was used to immobilize 5 µg of proteins onto the PVDF membrane prior to conducting the blocking process and subsequent incubation with the primary antibody. The same primary and secondary antibodies were used as in the western blot experiments. The additional primary antibodies were: anti-Toll-like receptor 7 TLR7 (ab24184; Abcam, Cambridge, UK), anti-Caspase 3 (ab13847; Abcam, Cambridge, UK), anti-Caspase 8 (ab108333; Abcam, Cambridge, UK), anti-BID (ab62469; Abcam, Cambridge, UK), t-BID (ab10640; Abcam, Cambridge, UK), MyD88 (NB100-56698SS) (Novus Biologicals Bio-Techne, Minneapolis, MN, USA), NOS3 (sc-654), 8-OHDG (sc-66036), SOD1 (sc-101523), SOD2 (sc133134) (Santa Cruz Biotechnology, Dallas, TX, USA), and NOS3 (Ser1177) (#9571) (Cell Signaling Technology, Danvers, MA, USA).

Visualization for both western blots and antibody arrays was performed using enhanced chemiluminescence. An imaging system equipped with a CCD camera (Omega Lum G, Aplegen, Gel Company, San Francisco, CA, USA) was used for signal detection and quantification was performed with Licor Image Studio Lite ver5.2. Three western blots were analyzed for each condition.

### 2.6. Live-Cell ROS Imaging

The hippocampal cells, isolated as previously described and destined for ROS imaging, were cultured for five days on a cover glass with a thickness of 85–115 µm exhibiting very low auto-fluorescence (Schott D 263^®^ M Glass #0, Thorlabs, Newton, NJ, USA) in a 6-well Corning culture plate. Imaging of intracellular reactive oxygen species (ROS) was conducted using chloromethyl 2′,7′-dichlorodihydrofluorescein diacetate (CM-H2DCFDA; Invitrogen, Waltham, MA, USA). This compound, CM-H2DCFDA, enters cells passively and non-invasively, where it becomes trapped inside the cell after hydrolysis and cleavage by nonspecific esterases, yielding 2′,7′-dichlorodihydrofluorescein (CM-H2DCF). When oxidized by ROS, such as hydrogen peroxide, CM-H2DCF transforms from a nonfluorescent state to a bright green, fluorescent form, 2′,7′-dichlorofluorescein (DCF). The chloromethyl variant of H2DCFDA, CM-H2DCFDA, shows enhanced retention within live cells compared to H2DCFDA. The cells in culture were treated with 5 μM CM-H2DCFDA for 30 min at a temperature of 37 °C. Following this, the cells were rinsed three times with Tyrode solution and the cover glass was removed from the 6-well plate, mounted in a Siskiyou perfusion chamber (Automate Scientific, Berkeley, CA, USA), and examined using a microscope equipped for epifluorescence (Nikon TS100, Sutter Instruments LS-17 xenon arc lamp), with fluorescence measurements taken at 488 nm using a Nikon SuperFluor ×20 objective. Then, the cells were immediately perfused with imiquimod solution (20 microgram/mL) using a ValveLink8.2 Perfusion Controller (Automate Scientific, CA, USA). The extra solution volume was recovered using an Instech P720 peristaltic pump (Instech, Plymouth Meeting, PA, USA). Cinnamon extract (1/1000 concentration) or plasma (1/1000 concentration) from various animal groups participating in the in vivo segment of the study was introduced to the cells five minutes prior to beginning the ROS imaging experiment. A digital live-cell fluorescence imaging system (InCyt Im2; Intracellular Imaging Inc., Cincinnati, OH, USA) equipped with a low-light CCD camera (Basler SCA 640-74) and a Lambda 10-B filter wheel controller (Sutter Instrument, Novato, CA, USA) was utilized to continuously capture and analyze images of various regions of interest (ROIs) for five minutes. Fluorescence curves were simultaneously generated by the system, corresponding to the fluorescence intensity within the cells. To calculate the rate of change in fluorescence, two distinct time points were chosen, before and after the acute addition of the treatment, and the respective difference in fluorescence levels was reported over time (ΔF·s^−1^).

### 2.7. Statistical Analysis

Data analysis was performed using GraphPad Prism 9, with the results expressed as the mean ± SEM and sample sizes detailed in the figure legends. Normality of distribution was assessed using the Shapiro–Wilk test. For data with a Gaussian distribution, Bartlett’s test was used to check for equal variance, while the Brown–Forsythe test was used for skewed data. Comparisons across more than two groups with normal distribution and equal variance were performed using one-way ANOVA, followed by Tukey’s or Sidak’s post-hoc tests to identify significant differences. When variances were unequal, the Brown–Forsythe and Welch ANOVA tests were applied, followed by Dunnett’s T3 for post-hoc analysis. For non-normal data, the Kruskal–Wallis test followed by Dunn’s test was used. Statistical significance is indicated by *, **, ***, and **** for *p* values of <0.05, <0.01, <0.001, and <0.0001, respectively, with ‘ns’ indicating non-significance.

## 3. Results

### 3.1. In Vivo Results

#### 3.1.1. IgG Deposition in Brain Tissue and Activation of TLR7/MyD88 Pathway

Hippocampal IgG deposition was documented in all lupus groups but less markedly in the CLC group (Figure 1B). A non-significant trend toward higher TLR7 expression was observed in the lupus group (Figure 1G). More importantly, the expression of MyD88 protein, a key adaptor in the TLR7 signaling pathway, was significantly enhanced in lupus mice compared to sham mice (Figure 1H). This increase was dampened in cinnamon-treated lupus mice (LC and CLC, *p* < 0.05, Figure 1H).

#### 3.1.2. Oxidative Stress Markers in Brain Tissue

A significant increase in the p-NRF2/NRF2 ratio was obtained in lupus mice compared to sham mice (Figure 1E,O). However, the ratio was decreased in the post-induction and preventive cinnamon treatment groups compared to the lupus mouse group (*p* < 0.05). In parallel, a significant increase in the p-FOXO3/FOXO3 ratio was observed in lupus mice compared to sham mice (Figure 1F,M) and this increase was significantly reversed in cinnamon-treated mice (CL and CLC). Notably, there were no significant differences observed in p-NOS3, NOS3, SOD1, and SOD2 expression between groups.

#### 3.1.3. Apoptosis Markers in Brain Tissue

**Figure 2:**
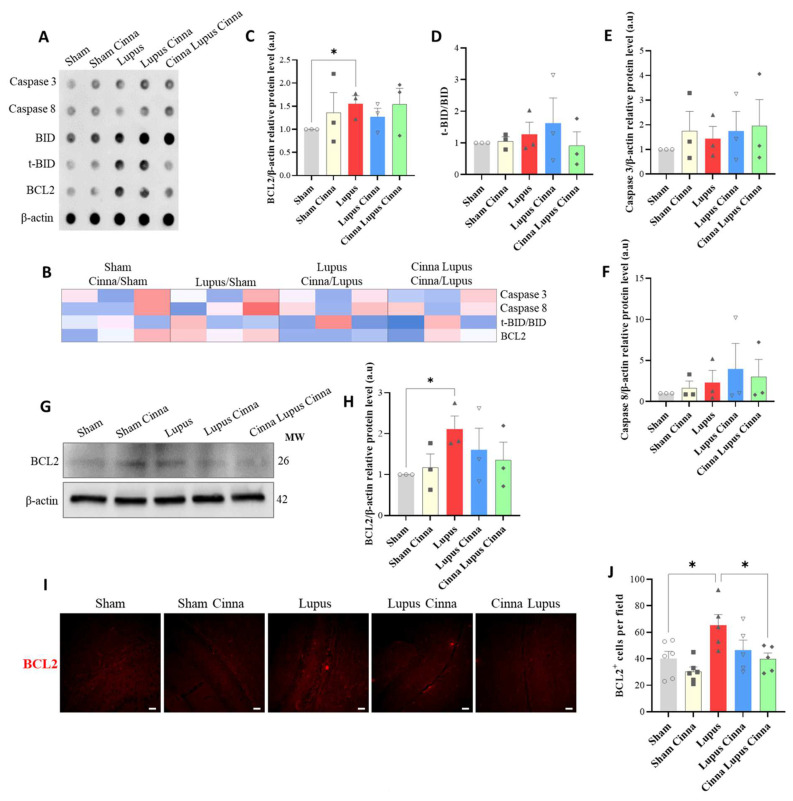
(**A**,**B**) Antibody array (**A**) with heatmap (**B**) of apoptotic markers for different groups. (**C**–**F**) Quantification of BCL-2/β-actin, t-BID/BID, caspase 3, and 8/β-actin antibody arrays. (**G**,**H**) Western blot and quantification of BCL-2. (**I**,**J**) Hippocampal BCL-2 immunofluorescence (scale bars 15 μm, magnification ×100) and quantification of BCL-2-positive cells. a.u.: arbitrary units. * *p* < 0.05.

BCL2 expression was higher in lupus mice (*p* < 0.05). Cinnamon treatment did not significantly alter BCL2 expression, although a trend toward lower protein expression was noted in the preventive cinnamon group in the western blots (Figure 2C,H). Immunofluorescence study of cellular BCL2 showed that this decrease was significant in the CLC group (Figure 2J). No significant modifications were found in caspase 3 and 8 expression and the t-BID/BID ratio between groups.

### 3.2. In Vitro Results of Cell Cultures Treated with Plasma of Different Mice Groups

#### 3.2.1. ROS Imaging and Oxidative Markers

**Figure 3:**
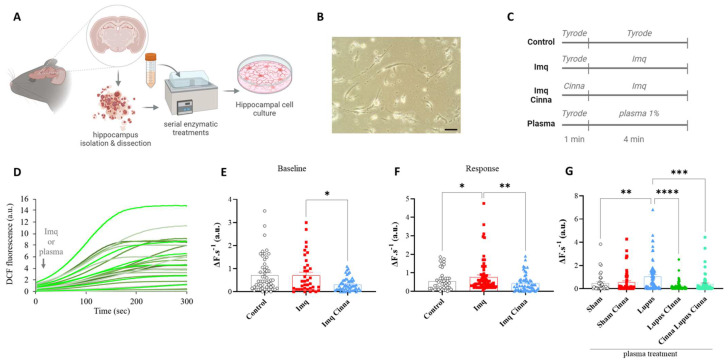
(**A**) Schematic representation of hippocampus isolation, enzymatic treatment, and culture. (**B**) Microphotography of cultured hippocampal cells (scale bar 10 μm, magnification ×200). (**C**) schematic representation of in vitro treatment protocol of cells with imiquimod, cinnamon followed by imiquimod, or the plasma of different mice groups. (**D**) 2′,7′-dichlorofluorescein (DCF) fluorescence variation after imiquimod or plasma treatment of hippocampal neurons. (**E**,**F**) Difference in fluorescence between groups at baseline (before imiquimod treatment) and after imiquimod treatment. (**G**) Difference in fluorescence between groups after treatment of hippocampal cells with the plasma of different mice groups. Cinna: cinnamon. Imq: imiquimod. a.u.: arbitrary units. * *p* < 0.05, ** *p* < 0.01, *** *p* < 0.001 and **** *p* < 0.0001.

**Figure 4:**
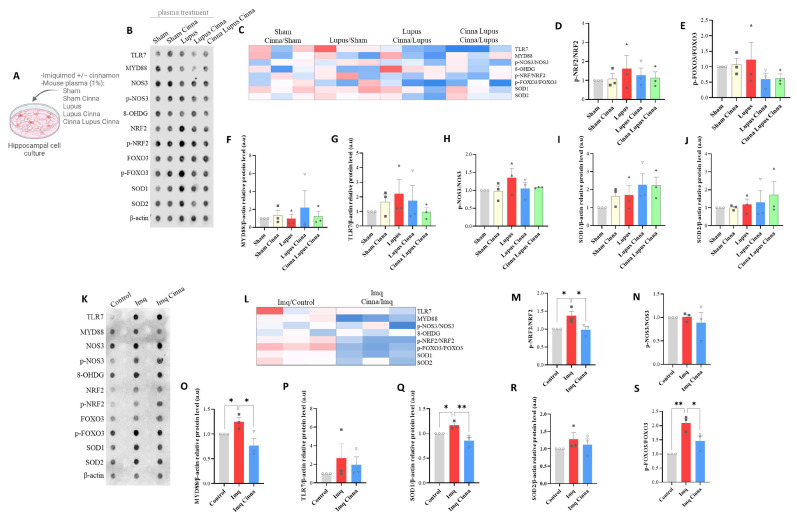
(**A**) Schematic representation of in vitro treatment of cultured hippocampal cells with imiquimod, imiquimod with cinnamon, or the plasma of different mice groups. (**B**–**J**) Antibody arrays, heatmap, and quantification of TLR7, MYD88, and oxidative stress markers in hippocampal cells treated with the plasma of different mice groups. (**K**–**S**) Antibody arrays, heatmap, and histograms of TLR7, MYD88, and oxidative stress markers in cultured hippocampal cells treated with imiquimod, cinnamon with imiquimod, or sham. a.u.: arbitrary units. * *p* < 0.05, ** *p* < 0.01.

ROS imaging revealed a significantly higher variation in fluorescence, reflecting higher production of ROS, after treatment of the cell cultures with lupus mouse plasma (Figure 3G). Compared to the lupus group, treatment with the plasma of the LC and CLC groups induced less ROS production (Figure 3G). Array and western blot analyses showed non-significant increases in the p-NRF2/NRF2 and p-FOXO3/FOXO3 ratios (Figure 4D,E) in cell cultures treated with the plasma of lupus mice.

#### 3.2.2. TLR7/MyD88 Pathway

Treatment of cell cultures with the plasma of lupus mice induced a trend toward increased TLR7 expression compared to that with the plasma of sham mice and non-significantly lower expression of TLR7 after treatment with the plasma of the cinnamon-treated groups. A similar profile was observed for MYD88 (Figure 4F,G).

#### 3.2.3. Apoptotic Markers

**Figure 5:**
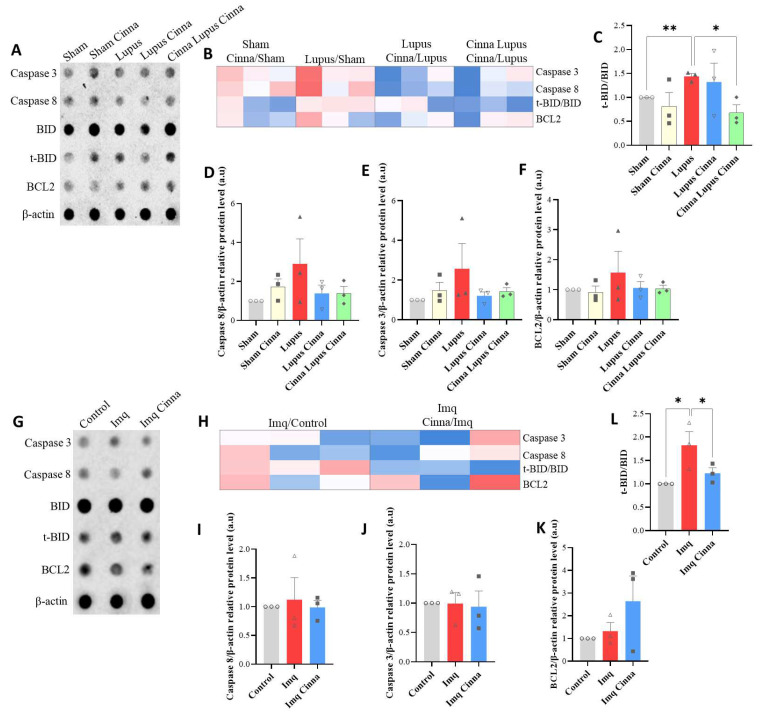
(**A**–**F**) Antibody arrays, heatmap, and quantification of apoptotic markers in cultured hippocampal cells treated with the plasma of different mice groups. (**G**–**L**) Antibody arrays, heatmap, and quantification of apoptotic markers in cultured hippocampal cells treated with imiquimod, cinnamon with imiquimod, or sham. * *p* < 0.05, ** *p* < 0.01.

After treating cell cultures with the plasma of different mouse groups, the t-BID/BID ratio was significantly higher with lupus mouse plasma, and this effect was dampened with the plasma of the CLC group (Figure 5C). Trends toward higher BCL2 expression, caspase3/β-actin ratio, and caspase 8/β-actin ratio were observed with lupus mouse plasma treatment, without reaching statistical significance (Figure 5D–F).

### 3.3. In Vitro Results of Cell Cultures Treated with Imiquimod or Imiquimod with Cinnamon

#### 3.3.1. TLR7/MyD88 Pathway

A trend toward increased TLR7 was observed in imiquimod-treated cells, while cinnamon seemed to buffer this increase (Figure 4P). More importantly, a significant increase in MyD88 expression was observed in imiquimod-treated cells. This increase was significantly reversed in cinnamon-treated cells (Figure 4O).

#### 3.3.2. ROS Imaging and Oxidative Stress Markers

Pre-treatment of cell cultures with cinnamon induced a significantly less important surge in ROS production and protected the culture milieu from oxidative stress induced by imiquimod (Figure 3E,F). A significant increase in the p-NRF2/NRF2 ratio was found in imiquimod-treated cells (Figure 4M). This increase was significantly reduced with cinnamon treatment. A similar pattern was observed for the p-FOXO3/FOXO3 ratio, which was significantly increased in imiquimod-treated cells and significantly decreased with the addition of cinnamon (Figure 4S). Moreover, SOD1 expression was enhanced with imiquimod treatment (*p* < 0.05), and this increase was significantly reversed with cinnamon treatment (Figure 4Q, *p* < 0.01). However, a non-significant increase in SOD2 expression was observed in imiquimod-treated cells. Notably, no significant modifications were found in the p-NOS3/NOS3 ratio.

#### 3.3.3. Apoptosis Markers

A higher t-BID/BID ratio was shown in cell cultures treated with imiquimod (*p* < 0.05), which was decreased with the addition of cinnamon to the milieu (Figure 5L). No significant variations were found in the expression of caspases 3 and 8 and BCL2 (Figure 5).

## 4. Discussion

To our knowledge, this is the first study to show that oral cinnamon supplementation alleviates oxidative stress and apoptotic markers in the brain of mice in imiquimod-induced lupus. The benefit effects of cinnamon were particularly evidenced with preventive intervention in three protocols (in vivo, in vitro treatment with the plasma of different groups, and in vitro treatment with imiquimod and cinnamon). These findings add new data about the neurologic damage pathways in lupus and highlight the neuroprotective effect of cinnamon added to the daily diet, mediated by its ability to rescue brain redox and apoptosis homeostasis in lupus. As our study was limited to dietary supplementation of cinnamon, future comparative studies should investigate the effects of other routes of administration of cinnamon.

While spontaneous murine lupus models are useful for understanding the immunogenetic basis in lupus, induced lupus models in wild strains help to elucidate the mechanisms by which environmental factors participate in lupus pathology. Two treatment arms (cinnamon supplementation starting two weeks before induction versus starting with induction) were evaluated in our study to stress the differential effect of the timing of cinnamon treatment initiation.

TLR-7 induction with imiquimod, a topical TLR-7 agonist, is the basis of the experimental lupus model in our study: wild-type mice subjected to epicutaneous application of imiquimod cream, three times weekly, developed lupus-like manifestations [19]. In this model, plasmacytoid dendritic cells (PDCs) redistribute from lymphoid organs to cutaneous tissue exposed to imiquimod, with enhanced expression of splenic *Ifna* and IFNα-stimulated genes. Anti-PDC antibody depletes PDCs and reduces anti-dsDNA titers induced by imiquimod [19]. New experimental and human data pinpoint the central position of the TLR7–MyD88 axis in lupus initiation and its blocking as a new therapeutic target [20]. It is interesting to note that hydroxychloroquine, a cornerstone in lupus management, has the ability to decrease TLR7 activation by acting on endosome pH [21].

The role of TLR7 in neuroinflammation is gaining interest in several models of brain injury [22,23,24]. However, TLR7 has been scantly investigated in the central nervous system in neuropsychiatric lupus. For example, one study of spontaneous lupus in NZB/W F1 mice demonstrated the central action of TLR7 in the brain of neuropsychiatric lupus and the benefit of its modulation [9]. Our study is the first to document TLR7 variation in the brain of an induced lupus model and its modulation by cinnamon supplementation.

Interaction between TLR7 and oxidative stress is complex. TLRs activate downstream proinflammatory pathways when they combine with the adapter molecule, MyD88, leading to overproduction of ROS [25]. In respiratory viral infections, TLR7 is associated with oxidative burst [26,27]. In monocyte cell cultures, co-stimulation of various TLRs, including TLR7, induces IL-1β and enhances oxidative stress [28]. Recently, increased ROS production and increased aortic inflammation were found in a murine model induced by a TLR7 agonist [29]. Interestingly, direct imiquimod treatment did not upregulate TLR7/8 in transformed (HSV) and normal endothelial cells but induced ROS after two hours, with a marked drop in the GSH/GSSG ratio after 12 h. [30].

SLE pathophysiology has proven to be influenced by oxidative stress, as documented by several markers. Our study has the peculiarity of exploring, in the same line as TLR7/MyD88 pathway activation, oxidative and apoptotic marker induction in the brain tissue and their modification by cinnamon supplementation. Our results show that these three pathways are intricately related in the pathophysiology of neurological damage in lupus, and they are simultaneously targeted by cinnamon treatment.

8-hydroxydeoxyguanosine (8-OHdG) predicts oxidative stress-induced DNA damage. Its levels in urine, plasma, and various tissues have been associated with SLE disease [31]. The cerebrospinal fluid (CSF) level of 8-OHdG was found to be a marker of brain damage in pediatric lupus with central nervous system lesions [32]. Only one study has linked a cinnamaldehyde-containing plant extract mixture to decreased 24 h urinary 8-OHdG levels in an experimental model [33]. In our study, 8-OHdG exhibited only a weak signal in the heatmap of the dot blot array and was not found to play an important role in the central nervous system injury of this model.

NRF2 is a transcriptional activator of many genes implicated in cellular redox homeostasis. NRF2 hyperactivation in the brain of old mice is considered to be a compensatory mechanism in the brain in an effort to equilibrate increasing ROS levels [34,35]. In a mild stress situation, nuclear NRF2 translocation activates the transcription of multiple of antioxidant enzymes as a protection against environmental factors [36] and disrupted homeostasis related to aging [37]. Furthermore, increased IgG deposition in the liver, heart, and brain is found in female *Nrf2^−/−^* mice with many clinical and biologic features of lupus [38]. In MRL/*lpr* (Old) mice, the protective effect of conjugated linoleic acid may be explained by its potential to enhance the activation of the NRF2 response [5].

The bark of *Cinnamomum aromaticum (cassia cinnamon)* is a source of cinnamaldehyde (CA), an NRF2 inducer in many cells, providing protection against electrophilic insult. Cinnamaldehyde induces an increase in the NRF2 protein half-life mediated by ubiquitination blockage, resulting in an increase in intracellular glutathione [39]. Our results shed a new light on the complex interaction between NRF2 and cinnamon in lupus: the p-NRF2/NRF ratio significantly increased in lupus mice while it was almost normalized in the preventive cinnamon group, indicating less oxidative stress (and thereby less compensatory activation of Nrf2) with preventive administration of cinnamon. This suggests that redox homeostasis was preserved by previous enrichment of the brain milieu by cinnamon components so that lupus induction in vivo or exposure of cells to the plasma of lupus mice in vitro failed to disrupt the redox homeostasis in brain tissue. This was paralleled by a trend toward less IgG deposition in hippocampal tissue.

FOXO3a, a member of forkhead box class O (FOXO) transcription factors, is implicated in oxidative stress resistance [40]. Evidence indicates that FOXO3 exhibits a regulatory role in immune-mediated diseases by inhibiting NF-κB [41]. In addition, the FOXO family seems to influence the neural cell cycle and physiology in mammals, clearance of ROS in the central nervous system, and axonal degeneration associated with aging [42].

Post-translational phosphorylation regulates the function of FOXO proteins. Phosphorylation by the serine/threonine protein Akt of FOXO3a inhibits many cellular functions targeted by FOXO3a [43].

Many studies suggest that unphosphorylated FOXO3a is the nuclear active form [40]. In one lupus experimental model, activated lymphocyte-derived apoptotic DNA stimulation induced the phosphorylation of FOXO3a and its cytosolic translocation and degradation. Glucocorticoid treatment in lupus significantly attenuated this induction of FOXO3a phosphorylation and rescues its function [40].

In our study, the p-FOXO3/FOXO3 ratio increased in the brain tissue of lupus mice, while cinnamon administration significantly attenuated the effect of TLR7 induction on the phosphorylation of FOXO3. Combined with the effect obtained on NRF2, these findings unfold the broad spectrum of cinnamon modulating activity on multiple oxidative targets, simultaneously with TLR7 attenuation in the lupus mouse hippocampus.

Nitric oxide synthases (NOSs) are responsible for NO production. Excessive NO can trigger neuronal death. Inversely, mice with macrophage NOS3 knockout have suppressed NF-*κ*B signaling and consequently neuroinflammation [44], suggesting that NOS3 mediates neuroinflammation [45].

NOS has been incriminated in lupus pathogenesis. Apoptosis in splenic lymphocytes of MRL/lpr mice was decreased by experimental suppression of NOS [46]. No significant variations in p-NOS3 were documented in our study, despite a trend toward a higher p-NOS3/NOS3 ratio observed upon treatment with the plasma of lupus mice.

Superoxide dismutases (SODs) are key players in cellular ROS homeostasis. SOD1 expression is ubiquitous and regulated by transcription factors sensitive to oxidative stress [47]. Single-nucleotide polymorphisms of SOD2 were associated with lupus susceptibility in an Egyptian pediatric population [48].

The role of SOD1 in neuroinflammation was mainly studied in an amyotrophic lateral sclerosis (ALS) model in mutant mice with hyperactivation of interferon pathways [49].

Oxidative stress due to SOD1 deficiency induces a lupus phenotype in C57BL/6 mice and exacerbates hemolytic anemia in NZB mice [50]. Trans-cinnamaldehyde was proven useful to upregulating hippocampal SOD1 in mice with experimental cognitive impairment [51]. In our study, SOD1 expression variation was significant only for in vitro treatment with imiquimod and cinnamon, with a compensatory rise in imiquimod treatment.

Under severe oxidative stress, cell viability is compromised. Recent data show that apoptosis may be triggered by oxidative stress [6]. For example, modulating the SIRT1/FoxO3a/Nrf2 pathway may have cytoprotective and antiapoptotic effects [52].

Neuronal injury and apoptosis are linked to ischemic and inflammatory mechanisms in SLE [53] and may be associated with caspase pathways [54].

In one study, serum from MRL/lpr mice triggered apoptosis in cultured human brain microvascular endothelial cells [54], with increased gene expression of many caspases and BCL2-associated X protein [54].

In our study, chronic neuroinflammation in lupus mice triggered by chronic activation of the TLR7 pathway seemed to induce an increase in Bcl-2 expression in the brain tissue as a compensatory neuroprotective mechanism against chronic oxidative stress. Chronic administration of cinnamon, especially in the preventive arm, offered potent antioxidant protection and may have superseded the need for Bcl-2 overexpression. This is compatible with other data demonstrating the neuroprotective properties of A-type cinnamon procyanidin mediated by regulation of the P38MAPK/P53/BAX pathway [55]. The Bcl-2 family includes a variety of inhibitor and activator proteins that regulate apoptosis [56]. Bcl-2 is a bridge between oxidative stress and apoptosis [57], and some polymorphisms are associated with lupus [58]. Anti-apoptotic Bcl-2 action involves binding to proapoptotic proteins, but it also influences cellular redox homeostasis independently of its regulation of apoptosis [59]. Bcl-2 expression increases cellular adaptation to oxidative stress and may affect mitochondrial generation of ROS [60]. Inversely, oxidative stress is a strong inducer of the Bcl-2 pathway [61].

In our study, the t-BID/BID ratio was increased in vitro after exposure of hippocampal cell cultures to lupus mouse plasma, whereas the CLC group plasma induced a significantly lower increase in the t-BID/BID ratio. Bid cleavage is implicated in ischemic and traumatic brain cell death [62,63] and contributes to cerebral damage induced by D-galactose [64].

## 5. Conclusions

Our study shows for the first time the dysregulation of redox homeostasis and apoptosis markers in brain tissue with concomitant activation of the TLR7/MyD88 pathway in an imiquimod-induced lupus model. Cinnamon supplementation, especially in the preventive arm, may calm down the innate immune activity in the brain tissue of lupus mice by rescuing redox and apoptosis homeostasis. The neuroprotective properties of cinnamon appear to be a promising preventive and therapeutic strategy in lupus, which may be tested in clinical studies as an add-on to the classical treatment armamentarium in lupus.

## Data Availability

Data is contained within the article.
